# Chefs in Future Integrated Healthcare – Current State and Innovation Needs: A First Overview of the NECTAR Project (aN Eu Curriculum for Chef gasTro-Engineering in Primary Food Care)

**DOI:** 10.5334/ijic.6436

**Published:** 2022-04-19

**Authors:** Marjolein Winters, Valentina Wagner, Roberta Patalano, Sonja Lindner, Serena Alvino, Regina Roller-Wirnsberger, Heidemarie Müller-Riedlhuber, Sandra Pais, Matilde Borriello, John Farrel, Geertrui Vlaemynck, Martijn van Gemst, Bart Geurden, Lobke Van den Wijngaert, Edwig Goossens, Maddalena Illario, Carolin Herzog

**Affiliations:** 1 Odisee University of Applied Sciences, Department of Business Management, Warmoesberg 26, 100 Brussels, BE; 2 Medical University of Graz, Department of Internal Medicine, Research Group for Old Age Medicine and lifelong Health, Auenbruggerplatz 15, 8036 Graz, AT; 3 Dipartimento di Medicina Clinica e Chirurgia, Sezione di Endocrinologia, Università Federico II di Napoli, via S. Pansini n.5, 80131 Naples, IT; 4 SI4LIFE scrl, Via Gramsci 1, 16126 Genoa, IT; 5 WIAB, Viennese Institute for Labour Market and Education Research, Leebgasse 46/1, 1100 Vienna, AT; 6 University of Algarve, Faculty of Medicine and Biomedical Sciences, Campus de Gambelas, 8005–139, Faro, PT; 7 IPSEOA Marco Polo, via Sciaccaluga 9, 16147 Genoa, IT; 8 EIP on AHA Reference Site Collaborative Network (RSCN) Round Point Schuman 11, B1040 Brussels, BE; 9 Flanders Research Institute for Agriculture, Fisheries and Food, ILVO, Department Technology and Food Science, Brusselsesteenweg 370, 9090 Melle, BE; 10 Center for Primary Food Care - Primary npo, Vissersstraat 1, 3000 Leuven, BE; 11 Faculty of Medicine and Health Sciences, Centre for Research and Innovation in Care (CRIC), University of Antwerp, Universiteitsplein 1, 2610 Wilrijk, BE; 12 ScC (Taste Center Consult & Coaching) LiveEatTaste, Heirbaan 277A, 2070 Burcht, BE; 13 School of Gastrologic Sciences & Primary Food Care, Center for Gastrology – van Rhay cvba, Vismarkt 10c, 3000 Leuven, BE; 14 Dipartimento di Sanità Pubblica, Università degli studi di Napoli Federico II, via S. Pansini n.5, 80131 Naples, Italy; 15 UOS Ricerca e Sviluppo, Azienda Ospedaliera Universitaria Federico II, via S. Pansini 5, 80131 Naples, IT

**Keywords:** integrated health care systems, chef, vocational education and training (VET), interprofessional, food, nutrition, malnutrition

## Abstract

People in need of care, chronic or acute, often present problematic food intake and special nutritional needs. Integrated, person-centred and pro-active food and nutritional care delivery has been proven effective for people in health care. However, skills mismatches have been reported in different professions involved, which also applies to the role of chefs in healthcare. The EU funded project NECTAR aims at closing this gap by creating a new job profile, called Chef Gastro-Engineering (CGE). The current publication summarizes the status quo in hospitals and gives a perspective on the future role of chefs in integrated healthcare delivery.

## Context and aim

Change of appetite, decreased food intake, unintended weight loss and psychological stress are all considered independent risk factors as well as symptoms of malnutrition [1]. If not managed adequately, malnutrition leads to multiple negative health outcomes and a significant economic burden [2,3,4]. According to a recent meta-analysis [5], the prevalence of malnutrition is higher in hospitals (28%) than residential care (18%) and community setting (8,5%). Although it represents a common condition, malnutrition may remain unrecognized for a long period of time. Especially hospitalized elderly patients are not always concerned about decreased food intake or fully realize their nutritional status and loss of body weight [6]. Moreover, reduced food intake during hospitalization is associated with variables related to both patients’ condition (e.g. clinical, physical) and factors related to the quality of hospital food itself [7].

The present paper reflects the role of chefs in the integrated food and nutrition care team in hospitals. Special attention is drawn on political frameworks underlining new perspectives and needs in the field of healthcare provision and the education of professionals involved in the care pathway. Therefore, this paper highlights an innovative approach to food and nutrition care provision including a new professional profile of chefs in healthcare.

## Current situation of interprofessional nutrition care in hospitals

Delivering adequate nutritional care for a large group of people characterized by complex care needs requires audited standards, education and training for smooth communication between care professions, but also coordination and integration between different stakeholders working in an institution and outside. This is based on dimensions that reflect the culinary (primary food care) and the mixed culinary-clinical (collaboration primary food care/secondary nutritional care) of interventions [8]. Currently, effective food and nutritional care in hospitals includes nurses screening patients on admission, monitoring them and ensuring food intake, dieticians or registered nurses, depending on the care setting, assessing nutritional needs and communicating individual goals to medical doctors. However, there are still gaps in the care pathway like lack of nursing time, knowledge of balanced diets and constituent food groups, inadequate communication, trust, quality of food and beverages, and respect within care teams [9]. Additionally, the personal tastiness of meals and eating and chewing abilities are often neglected, stretching the importance of the chef in a care team. This opens a bigger picture of effective food and nutrition care delivery in health care, chefs becoming a potential part of an integrated food and nutrition care delivery model [10].

This horizontal integration between professionals and care sections of institutions allows person-centred and proactive care delivery especially for those most vulnerable, who are in need for tailored food and nutrition care approaches. Although modern European society has created many services to support vulnerable populations [11], these services are divided into organizational clusters, managed, and delivered in an uncoordinated and isolated manner without considering the chef’s perspective.

## European educational frameworks and political strategies fostering the integrated care model

The World Health Organization recently published two strategies promoting interprofessional care approach where health workers work together across organizational boundaries [12,13]. Interprofessional teamwork differs from the multidisciplinary approach, aligning and integrating skills of different professions on a shared competence base, making all members of the team equally important for success of care provision [14]. This improves care for an ageing population [15,16] and addresses specific food needs of persons and/or malnutrition [17]. However, this requires adequately trained staff and clear skills distribution of care professions to assure successful collaboration and favourable outcomes.

Although the European Skills, Competences, Qualifications and Occupations inventory describes occupational profiles, skills, competencies and qualifications of nutritional healthcare workers [18,19,20,21,22], the healthcare workers report shows a high rate of skills mismatches because of increased demand of more complex and changing working environments due to reforms of healthcare systems toward more integrated and personalised care [23]. This also includes the role of chefs in healthcare and may be observed in all EU member states (MSs). Chefs in healthcare should be considered as part of an integrated food and nutritional care team as they address food preferences, organoleptic food qualities, alter recipes and menus and encourage food consumption and calorie intake [24,25].

Standardization of health workers’ professional profiles, adaption of professionals’ training and alignment of professional inputs along persons’ journeys through healthcare systems are needed to address the interprofessional care delivery demanded in those policy documents.

Throughout Europe, tools allow quality cross-national control of Vocational Education and Training (VET), support national MS in implementing health workforce training on national level [26] and are the strategic framework for European cooperation in education and training [27]. EU frameworks like the European Quality Framework (EQF) or the European Quality Assurance in Vocational Education and Training [28,29] build the ground to go beyond the current service delivery and allow future developments in health workforce development.

## Integrated food and nutrition care in institutions

Integration of healthcare improves patient experiences and serves better outcomes of care delivery and effective healthcare systems [30,31]. One challenge is the highly context-sensitivity and the myriad among care providers, that integrated care is a “processual concept” only, rather than a consistent and designated model [31,32,33].

**Figure 1:**
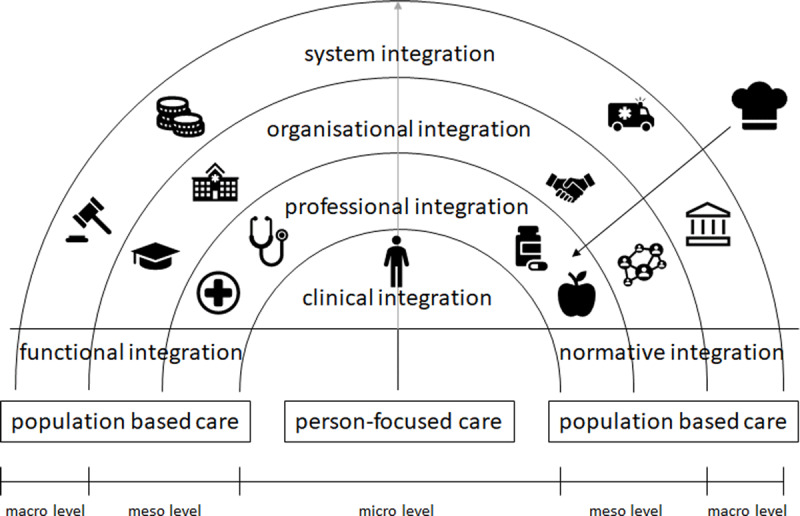
Adaption of Conceptual Framework of Integrated Care considering Chefs. Legend: Figure 1 shows conceptual framework for integrated care by Valentijn et al., 2013 (32) modified to show the role of the chef/cook in an integrated nutritional care team.

To overcome these barriers, Valentijn et al. have developed a comprehensive conceptual framework for integrated care. ***Figure 1*** visualises this model, high-lightening the future role of chefs in integrated food and nutritional care team [34]. In practice, integrated care ideally permeates the clinical level, which puts focus on the person on a micro level, up to the professional level that enables coordination of services among different care professions [35]. For a horizontal integration of chefs, clear definitions of roles, responsibilities and principles are needed. Organizational integration facilitates integration of chefs in healthcare systems through inter-organizational relationships (e.g. educational institutions, workplaces, cooperation). System integration, on macro level, defines structures, policies, and governance of different care institutions (e.g. access to healthcare system, financial status of countries, legal structure).

This framework distinguishes functional from normative integration. Functional integration answers the question “who does what” and covers the tasks, activities, competencies, processes and tools necessary to provide coordinated care, while normative integration involves the context of care in terms of norms and values that guide care provision and daily demand [34]. In order to provide a key characteristic of integrated care, not only formal integration of structures and organizations need to be considered, but also an interdisciplinary way of working beyond professional silos [36,37].

As may be seen from ***Figure 1***, the rainbow model is very useful to reflect all dimensions of involvement of chefs in healthcare, from community to institutionalized care provision. To illustrate possible practice-based dimensions of this innovative approach to integrated food and nutrition care delivery, authors have collected hands-on duties for chefs possibly included in the future professional profile (***Table 1***).

**Table 1:** Examples for possible real-life Integration of Chefs in Healthcare.


LEVEL OF EFFECTIVE INTEGRATED FOOD AND NUTRITION CARE	CLINICAL INTEGRATION	PROFESSIONAL INTEGRATION	ORGANIZATIONAL INTEGRATION	SYSTEM INTEGRATION

Person-centered care	Chefs carrying out taste steering assessments with patients on taste disturbances and create solutions for taste deterioration; Chefs talking with patients about their satisfaction with intervention outcomes in cooperation with other health professionals	Chefs attending interprofessional team meetings and discussing treatment options with other professions in the nutritional care team	Chefs working together with educational institutions to educate chefs about nutrition and health Chefs working on promotion of workplace health, offering healthy cooking classes for employees	Chefs advocating for a high quality cooking process to improve the nutritional value of meals for patients with needs

Population-based care	Chefs creating cooking books for adapting meals and recipes for people with specific nutritional needs	Chefs cooperating with dieticians in developing baseline menus for people with specific needs	Chefs working together with insurance companies to promote cooking classes as social prescribing	Chefs working together with their professional association in order to promote healthy meals for the population and nutritional education for chefs


Legend: Table 1 illustrates practice-based examples, how chefs will be integrated in food and nutrition care delivery in healthcare through their new occupational profile developed during the ongoing EU-funded project “Nectar” (Grant agreement number 621707). As may be seen from the table, chefs’ involvement includes functional as well as normative aspects of integration. The profile developed will be based on learning outcomes during the training process, which include a comprehensive set of knowledge, skills and attitudes enabling chefs to become an integral part of the care team.

## Discussion and reflection

The future needs and perspectives outlined in this paper are targeted by the project NECTAR: aN Eu Curriculum for chef gasTro-engineering in primAry food caRe (*http://www.nectar-project.eu*). Funded by the EU Erasmus+ program, NECTAR targets skills panorama for chefs in healthcare. All standards outlined in this publication will be addressed and included into the new job profile of CGEs to allow a functional integration.

One strength of NECTAR is the participation of European stakeholders, defining the new job profile, competence list and learning outcomes for CGEs. The project has been designed on a co-creation design-approach for sustainable service innovation and value creation during change management [38]. During the development of the profile and competencies, all partners from different professional sectors in food and healthcare contribute content according to relatively strict submission requirements, so that contributions may be categorized as having fixed contribution and aligned in structured methodology [39].

Another strength is the implementation of training programs arising from the desktop work done during the project. Underpinned by a strong evaluation framework, which will allow direct comparison of program designs through Europe, all educational pilots will address the same European framework, but are offered from cooking school to university level.

One of the challenges of NECTAR is the different EQF entry levels of chefs after their basic qualification across Europe. The project has designed tools to address these differences and to allow scaling-up of the project results in every EU MS.

Drawbacks of the project are that the new curriculum and program delivered will not allow to measure impact on healthcare quality already during the project phase and that the impact of functional integration on organisational structures and on system level is not measurable.

## Conclusion

Evidence-based research points out that food and nutrition should become a cornerstone in future institutional healthcare delivery [40]. Although the EU has various frameworks and strategies for quality assurance, transferability and transparency for VET for professionals involved into that new care model [27,28,29,41], there is a skill gap for chefs in healthcare. Therefore, a high-standard workforce education is needed for chefs, which addresses the demand of the labour market.

The project presented in this publication gives a key perspective and solution to this challenge. Closing skills gaps for chefs in healthcare will allow a functional integration of chefs in the care team and will contribute to promote tasty, healthy and safe food compositions adapted for healthcare, not only at the national health and policy level but also at regional, institutional and individual level. Results gathered during the project are scale-able due to the project design and will push Europe and its MSs at the forefront of change management towards integrated interprofessional healthcare delivery.
